# New HSP27 inhibitors efficiently suppress drug resistance development in cancer cells

**DOI:** 10.18632/oncotarget.11905

**Published:** 2016-09-08

**Authors:** Jörg C. Heinrich, Sainitin Donakonda, V. Joachim Haupt, Petra Lennig, Yixin Zhang, Michael Schroeder

**Affiliations:** ^1^ Biotechnology Center, Technische Universität Dresden, 01307 Dresden, Germany; ^2^ B CUBE – Center for Molecular Bioengineering, Technische Universität Dresden, 01307 Dresden, Germany

**Keywords:** drug repositioning, drug repurposing, HSPB1, BVDU, binding site

## Abstract

Drug resistance is an important open problem in cancer treatment. In recent years, the heat shock protein HSP27 (HSPB1) was identified as a key player driving resistance development. HSP27 is overexpressed in many cancer types and influences cellular processes such as apoptosis, DNA repair, recombination, and formation of metastases. As a result cancer cells are able to suppress apoptosis and develop resistance to cytostatic drugs. To identify HSP27 inhibitors we follow a novel computational drug repositioning approach. We exploit a similarity between a predicted HSP27 binding site to a viral thymidine kinase to generate lead inhibitors for HSP27. Six of these leads were verified experimentally. They bind HSP27 and down-regulate its chaperone activity. Most importantly, all six compounds inhibit development of drug resistance in cellular assays. One of the leads – chlorpromazine – is an antipsychotic, which has a positive effect on survival time in human breast cancer. In summary, we make two important contributions: First, we put forward six novel leads, which inhibit HSP27 and tackle drug resistance. Second, we demonstrate the power of computational drug repositioning.

## INTRODUCTION

Heat shock proteins (HSPs) are evolutionarily conserved molecules synthesized by cells in response to heat or chemical stress. Acting as molecular chaperones, HSPs protect cells from environmental stress damage by stabilizing native folding of proteins. In addition, they help to sequester severely damaged proteins for degradation. HSPs are found to be overexpressed in a wide range of cancers. Members of the HSP family have been implicated in cancer resilience by promoting tumor cell proliferation as well as inhibiting cell death pathways. HSP27 in particular is emerging as a promising target in cancer therapy.

### Heat shock protein HSP27 as a cancer therapy target

HSP27 (Heat shock protein beta-1; HSPB1) is constitutively expressed in many cell types and tissues at specific stages of development and differentiation. In malignant cells, HSP27 expression correlates with the oncogenic status of the cell and has a role in their tumorigenicity. HSP27 is up-regulated across many types of cancer; including breast [1], colorectal [2], liver [3], tongue [4], bladder [5], eye [6], ovarian [7], and pancreas [8]. HSP27 is not just up-regulated in cancer cells, but it is specifically implicated in the development of drug resistance. Kuramitsu *et al.* show that increased HSP27 expression is related to higher rates of Gemcitabine resistance in pancreatic cancer cells [9]. In multiple myeloma, Chauhan *et al.* report that cells resistant to dexamethasone (Dex) overexpress HSP27 and that Dex-resistance can be overcome by inhibition of HSP27 [10]. For bladder cancer, Kamada *et al*. found that HSP27 overexpression is related to paclitaxel resistance and increased UMUC-3 cell growth [5]. Zhang *et al*. identified up-regulation of HSP27 in the doxorubicin-resistant, ERp29 over-expressing MDA-MB-231 breast cancer cell line [11]. Similar findings regarding drug resistance apply to prostate, pancreatic, and ovarian cancer [7, 12].

In summary, HSP27 overexpression is documented in over 10 cancer types and has been linked to resistance to more than 10 different cytotoxic agents [1, 5, 6, 9, 10, 13]. Therefore, Mori-Iwamoto observed that silencing of HSP27 reduces drug resistance [14]. Interestingly, there is evidence for the converse: In testis cancer, Richards *et al*. found HSP27 at low levels and observed an unusually good response to chemo- and radiation therapy [15]. As a conclusion, many authors agree that targeting HSP27 is a promising anti-cancer strategy.

### Targeting HSP27

For head and neck squamous cell cancer, glioma, breast and lung cancer stem cells there is evidence of a positive effect after inhibition of HSP27. Silencing of the HSP27 gene led to decreased metastatic behavior of human head and neck squamous cell cancer cells *in vitro* [16]. In glioma, the inhibition of HSP27 alone or in combination with a pAKT inhibitor has been described as a promising therapy approach in SPARC-induced glioma cells [17]. HSP27 has been described as a target in breast cancer therapy and the role of HSP27 in the maintenance of breast cancer stem cells was pointed out by Wei *et al*. [18]. The combination of HSP90 and HSP27 inhibitors to enhance the suppressive effect in breast cancer stem-like cells was proposed by Lee *et al.* [19] and decreased survival of lung cancer stem cells – otherwise resistant to chemotherapy – has been demonstrated by Hsu *et al*. using Quercetin to down-regulate HSP27 expression [20].

### HSP27 and apoptosis

HSP27 is such a promising target, as it protects cells against apoptotic cell death triggered by a variety of stimuli including hyperthermia, oxidative stress, staurosporine, Fas ligand and cytotoxic drugs [21]. It regulates apoptosis through interaction with STAT3 [22], CYC1 (Cytochrome c-1) [23], and CASP3 (Pro-Caspase 3) [24]. In previous work on target identification, we have shown that the herpes drug BVDU binds to HSP27. BVDU restores apoptosis by inhibiting the interaction of HSP27 with pro-apoptotic proteins. Our experiments showed that with BVDU binding to HSP27, there was less AKT1, CASP3, and CYC1, respectively, bound to HSP27. These findings confirmed the inhibitory effect of BVDU on HSP27 and its activating effect on apoptosis [25].

In summary, HSP27 regulates apoptosis and is linked to drug resistance in numerous cancers. Thus, modulating the activity of HSP27 is a promising approach to tackle drug resistance in cancer [12]. Our goal is to deploy a computational pipeline to discover selective, efficient, and experimentally validated HSP27 inhibitors to counteract resistance development in cancer chemotherapy.

## RESULTS

### A computational drug repositioning pipeline to predict HSP27 binders

Computational drug repositioning identifies novel targets and indications for existing drugs using bioinformatics databases and algorithms. Here, we pursued a structural approach, which exploits similarities between binding sites in otherwise unrelated target proteins to predict novel binders. Concretely, in previous work [25] we discovered a surprising connection between cancer and herpes, between HSP27 and a viral thymidine kinase (VTK). A 3D structural model of HSP27 and a crystalized structure of VTK showed a similar binding site with five identical residues in exactly the same geometric confirmation (see Figure 2A). Two of these residues are phenylalanines, which can coordinate aromatic rings of ligands in a very stable sandwich-like complex (pi-stacking). In [25], we showed computationally and experimentally that this shared binding site is capable of binding the ligand BVDU. Therefore BVDU is not only an anti-viral herpes drug, but also a candidate cancer drug. In this work, we generalize this hypothesis. If one VTK ligand binds HSP27, so may others.

**Figure 1:**
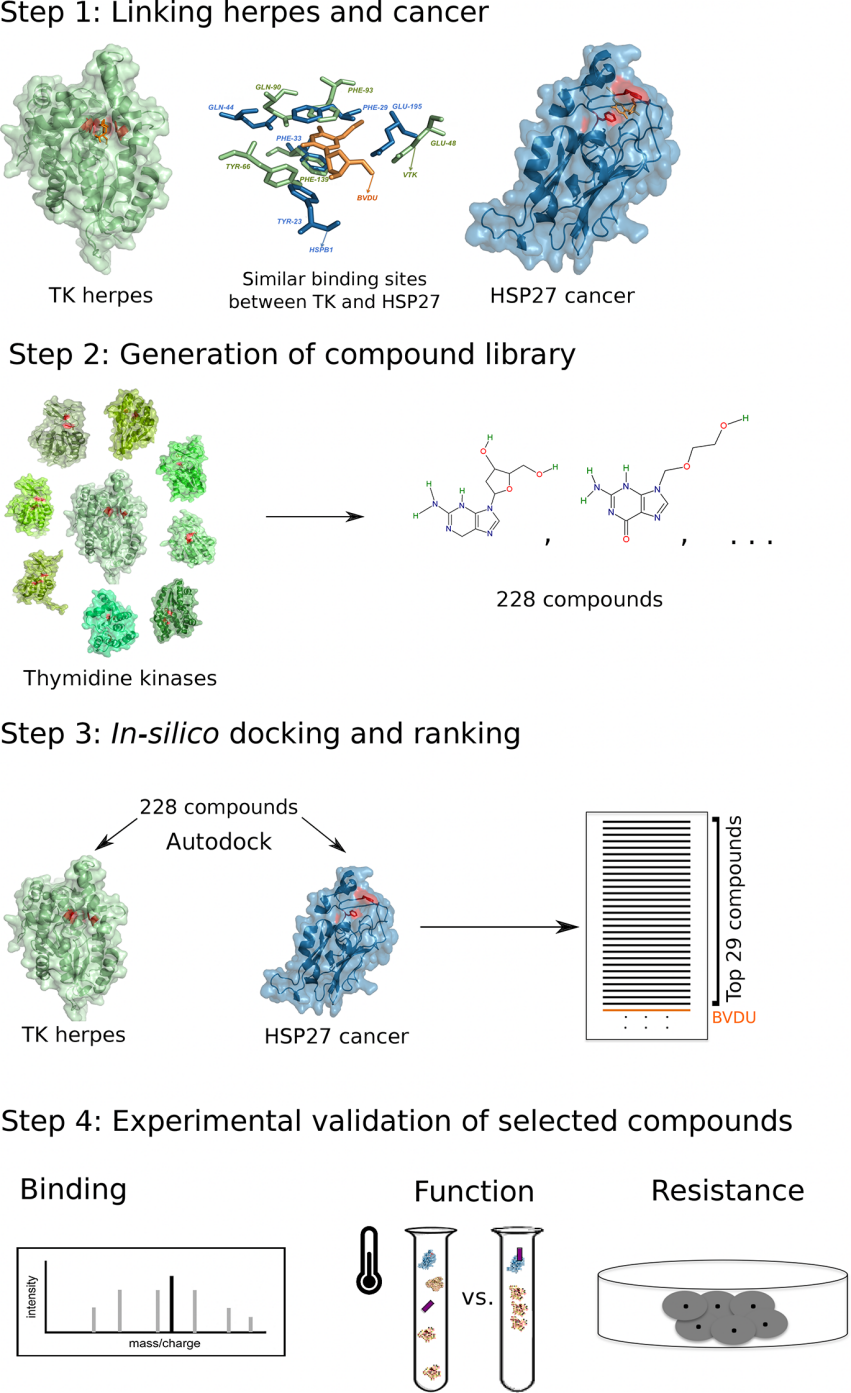
Computational drug repositioning pipeline to predict HSP27 binders Step 1: viral thymidine kinase and HSP27 share a binding site. Step 2: The potency of 228 thymidine kinase ligands to bind HSP27 is assessed with docking. Step 3: 29 of these ligands bind *in silico* better than the known binder BVDU. Step 4: Experimental validation of six ligands.

We tested this hypothesis through the following computational pipeline: We collected 115 ligands binding viral thymidine kinases and further expanded this set to 228 ligands considering non-viral thymidine kinases (Figure 1, step 2). Next, we tested ligand binding computationally by docking ligands into the thymidine kinase and the HSP27 pocket, respectively (Figure 1, step 3). Since our goal is an improvement over the known HSP27 inhibitor BVDU, we kept only those 29 ligands, which obtained better docking scores than BVDU. Finally, we selected six ligands for experimental validation (Figure 1, step 4).

**Figure 2:**
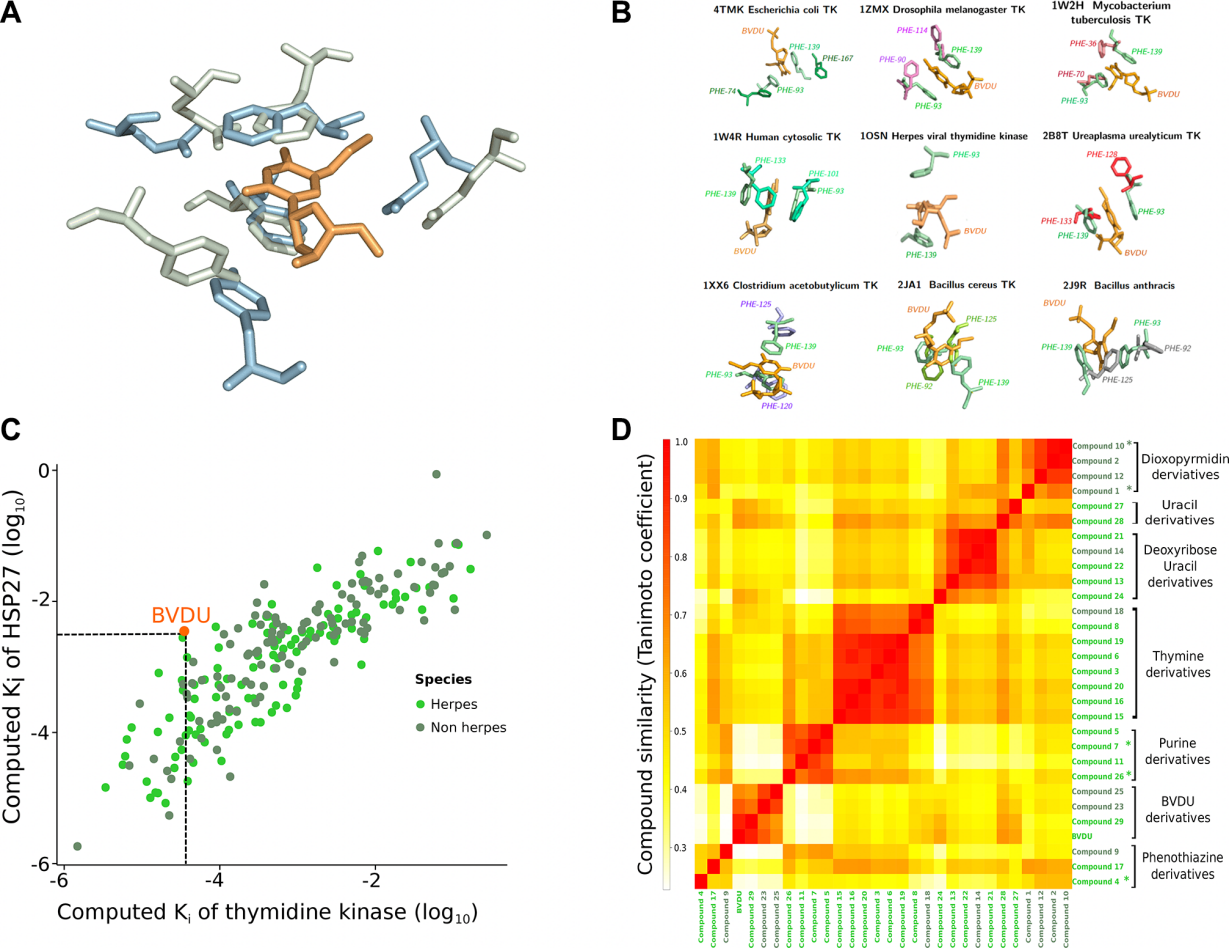
VTK and HSP27 (**A**) Viral thymidine kinase (green) and HSP27 (blue) share a similar binding site with five residues of very similar conformation. BVDU (orange) binds both VTK and HSP27. (**B**) Eight non-viral thymidine kinases (grouped around the central viral thymidine kinase) share a similar binding site to VTK. Overall, there are 228 ligands binding the viral and the non-viral TKs. (**C**) *In silico* docking of these 228 ligands against VTK and HSP27. The docking scores correlate (0.84, *p-value* < 10^−1^^6^). 29 ligands have higher computed affinities than the known binder BVDU. (**D**) One of the 29 ligands is closely related to BVDU, but the vast majority is chemically dissimilar.

### Binding site similarity between HSP27 and VTK

Consider Figure 2A. At the source of the computational drug repositioning pipeline is a shared binding site between a herpes thymidine kinase and HSP27. Strikingly, five residues are geometrically in the same arrangement. The two key residues are two phenylalanine residues, whose rings can coordinate the BVDU ring in a sandwich-like structure involving pi-stacking. Additionally, the other three residues mediate characteristic interactions.

### 228 thymidine kinase ligands may bind HSP27

Our drug repositioning hypothesis is that the above binding site similarity implies that ligands of thymidine kinases may bind HSP27. We collected TK binders in two stages. First, we obtained 115 ligands by retrieving herpes thymidine kinases from UniProt [26] and their ligands from BindingDB and TTD [27, 28]. We further expanded this set by considering non-herpes thymidine kinases. To avoid the introduction of too much noise, we inspected the binding sites of the non-herpes thymidine kinases. Thus, we collected non-herpes thymidine kinase sequences from UniProt and mapped these to PDB, obtaining 12 structures. Figure 2B shows the eight structures that have a similar binding site to VTK, which is placed at the center of Figure 2B. The structures cover bacteria, but also fruit fly (Drosophila) and human. For these eight structures, we found another 113 ligands, so that there are 228 ligands in total.

### Docking scores of VTK and HSP27 correlate

Next, we docked these 228 ligands against the VTK and the HSP27 binding sites, respectively. Figure 2C shows the computed binding affinities as scatter plot on a log scale. If the scores perfectly agree, there is no need to dock against both binding sites. If they disagree strongly, then the binding site similarity is too weak. However, we find a good agreement with a statistically significant correlation of 0.84 at a *p-value* of less than 10^−16^. As a key step we selected now those ligands, which show a higher computed binding affinity than BVDU in both binding sites. BVDU (the known ligand of both) docked with an affinity of K_i_ = 10^−2.46^ on HSP27 and K_i_ = 10^−4.45^ on VTK. A total of 29 ligands achieved better affinities (see Supplementary Figure S1).

### Most of the 29 lead HSP27 inhibitors are new

Often derivatives of ligands have similar or even better binding properties than the original drug. One could expect that the 29 ligands, which are better than BVDU, are direct derivatives of BVDU. However, they are not. To understand how the 29 compounds relate to each other structurally, we used chemical fingerprints, which represent key features of the compound structures and which make them directly comparable. Figure 2D shows these pairwise similarities as a heat map. The red rectangles in Figure 2D indicate groups of structurally related compounds. Overall, there are six groups of compounds: BVDU-, thymidine-, thymine-, deoxyuridine-, guanine-, and furo-pyrimidinone-derivatives, as well as a single phenothiazine, and a pyrimidine piperazine. Interestingly, only three compounds are direct derivatives of BVDU. However, the deoxyuridine-, thymidine-, and thymine derivatives can be related to BVDU. There is a progression starting from BVDU via the compounds 6 and 16 to the thymidine derivatives. While the heat map in Figure 2D grouped compounds 6 and 16 with other thymidine derivatives, the Supplementary Figure S2A shows that they comprise the bromovinyl group and can therefore be considered BVDU derivatives. In a next step, the thymidine derivatives are structurally related to the thymine derivatives. These structural changes and variations are not obvious. First of all, starting from BVDU one would expect the bromovinyl group of BVDU to play a critical role in binding to Hsp27. However, considering Supplementary Figure S2A, the transition from BVDU derivatives via compound 6 and 16 to the thymidine derivatives shows that the bromovinyl group can be replaced by a methyl group. This replacement goes hand in hand with a best rank of 23 for the BVDU derivatives to 3 for the thymidine derivatives. In the next step from thymidine derivatives to thymine derivatives, the deoxy sugar is replaced by a linker and the triazole, which mimics the amide group, is replaced by an amide group. Thus, the hydrophobic part of the compound is increased. Again, ranks improve to the top rank 1. Overall, the compounds in Figure S2A can be related to BVDU, although only indirectly via non-obvious changes. However, the 11 compounds in Figure S2B are unrelated to BVDU. Most importantly, there are guanine and furo-pyrimidinone derivatives. The guanine group comprises four closely related compounds and therefore sheds light on the possibilities for modification and its relation to activity. In contrast, the furo-pyrimidinone group provides little insight into the possibility for activity improvement. Finally, the group of single, completely unrelated compounds contains a sedative used in animals, acepromazine (compound 4).

### Selection of compounds for experimental validation

Based on structural considerations, on position in the ranking, and on availability, we chose six compounds for experimental validation. As shown in Figure 2B and Figure S2A the thymine derivatives 1 and 10 are indirect derivatives of BVDU, which are the most distant to BVDU and which scored well in the ranking (1 and 10). Originally, these compounds were developed as human mitochondrial thymidine kinase inhibitors [29].

Among the compounds unrelated to BVDU, we chose the guanine derivatives 7 and 26, because of their ranking at the top and bottom of the list and because of the possibilities for later improvements of the compounds. For this latter reason, we did not choose any of the furo-pyrimidinones at this stage. Compounds 7 and 26 were developed originally as inhibitors of *Herpes simplex* [30, 31]. Finally, we selected one of the single compounds, acepromazine, as it is in use in animals as a sedative and antiemetic. Acepromazine is very closely related to chlorpromazine, an FDA-approved antipsychotic drug used in humans, which we added for this reason to the selection.

To validate the six compounds we subjected them to three different tests: First, we checked for binding to HSP27. Second, we investigated the impact of binding on HSP27′s function as chaperone. Third and most important, we tested whether and how strongly the compounds reduce resistance development in cancer cell lines. The results of these three experiments are summarized in Table 1 and discussed in detail below.

**Table 1:** 6 compounds with summary results

Compound name	Pubchem ID	CAS Nr.	Ref.	Original Mode of Action	HSP27		Inhibition of	
					Docking Rank	Binding Assay	Chaperone Function	Resistance Development
Chlorpromazine	CID2726	50-53-3		Antipsychotic drug	–	–	80 ×	+++
Acepromazine	CID6077	61-00-7		Sedative used in animals	4	yes	74 ×	+++
2-(4-butylanilino)-9-(2-hydroxyethoxymethyl)-3H-purin-6-one	CID387676	104715-80-2	(35)	Herpes Simplex thymidine kinase inhibitor	26	yes	69 ×	++/+++
2-[3-(1,2,2-trichloroethenyl)anilino]-3,7-dihydropurin-6-one	CID3007762	161363-17-3	(36)	Herpes Simplex thymidine kinase inhibitor	7	yes	53 ×	++
N-[(Z)-4-(5-methyl-2,4-dioxopyrimidin-1-yl) but-2-enyl]-9H-xanthene-9-carboxamide	CID44333373	1222812-38-5	(34)	Human mitochondrial thymidine kinase inhibitor	1	yes	49 ×	++
N-[(Z)-4-(5-methyl-2,4-dioxopyrimidin-1-yl) but-2-enyl]-2-(4-phenylphenyl)acetamide	CID44333760	1222781-87-4	(34)	Human mitochondrial thymidine kinase inhibitor	10	yes	61 ×	++
Indomethacin	CID3715	53-86-1		Negative control	–	no	–	–
BVDU (Brivudine)	CID446727	69304-47-8	(25)	Herpes Simplex thymidine kinase and HSP27 inhibitor	30	–	1 ×	+

Based on chemical class, position in the ranking, and availability, six ligands were selected for experimental testing. Four ligands were specifically developed as thymidine kinase inhibitors, one is a sedative in use for animals, and one is an antipsychotic in humans. Additionally, a negative control and BVDU are tested. The six lead ligands perform significantly better than BVDU. Five out of six bind to Hsp27 in the SpeedScreen, all six inhibit chaperone activity better than BVDU (50–80 times), and all six reduce drug resistance development significantly. Structures of the compounds are depicted in Supplementary Figure S2.

### Five out of six lead inhibitors bind to HSP27

The SpeedScreen technique (see Methods) allows differentiation between binders and non-binders because small molecules, whose weight is below 5 KDa, will not pass through Sephadex G-25 Spin columns unless they are bound to a bigger molecule such as HSP27. Only passing molecules can subsequently be detected by mass spectroscopy.

Using the SpeedScreen we tested the six lead compounds, the negative control, and BVDU. Consider Table 2: Five of the six lead compounds bind HSP27 at concentrations ranging from 0.04 to 0.89 μM. As expected, the negative control Indomethacin does not bind. However, unexpectedly, BVDU and chlorpromazine cannot be detected. Since the two subsequent experiments for BVDU and chlorpromazine are positive, and since BVDU has been independently shown to bind HSP27 with a pull down assay [25], we conclude that this negative result is not due to non-binding of the two compounds, but rather due to the limits of the experimental method.

**Table 2:** Five out of six lead compounds bind HSP27 in the SpeedScreen assay

CAS Nr.	Concentration detected by MS	HSP27 Binding
50-53-3	–	–
61-00-7	0.89 μM	yes
104715-80-2	0.22 μM	yes
161363-17-3	0.04 μM	yes
1222812-38-5	0.21 μM	yes
1222781-87-4	0.25 μM	yes
53-86-1	0.000097 μM	no
69304-47-8	–	–

### All lead compounds inhibit HSP27′s chaperone activity

**Figure 3:**
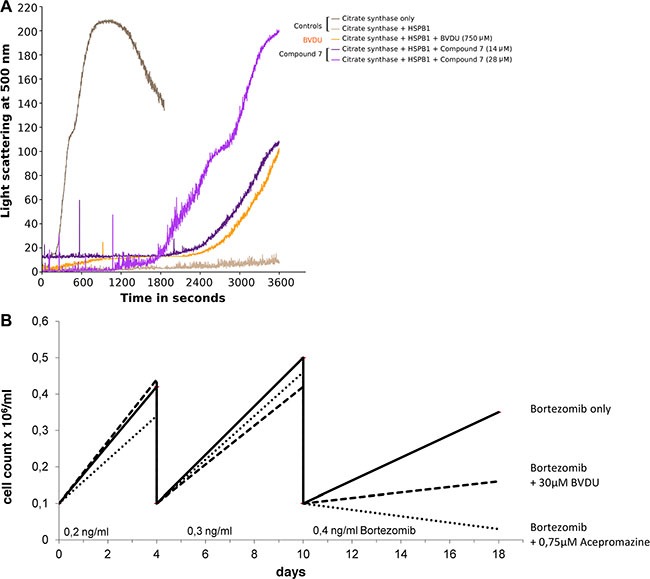
(**A**) HSP27 acts as chaperone for CS (citrate synthase + HSP27); compound 7 (14 and 28 μM) and BVDU (750 μM) inhibit HSP27 chaperone function. (**B**) Suppression of drug resistance versus Bortezomib was measured by comparing live cell counts over three passages comparing BVDU, a lead compound, and a control.

HSP27 acts as chaperone and helps client proteins to remain correctly folded under stress conditions. In the absence of functional HSP27, one such client protein, citrate synthase (CS), misfolds and forms aggregates when denatured by heat. Misfolded CS forms aggregates, so that light scattering detected with a spectrometer is an effective method to measure aggregation. Consider Figure 3A: The graph shows light scattering over time for two controls (CS on its own and with HSP27), with BVDU at 750 μM, and one lead compound (CAS 161363-17-3) at 14 and 28 μM, respectively. With CS alone the graph peaks after 15 minutes, whereas HSP27 keeps CS correctly folded over the whole course of an hour. BVDU (750 μM) and the lead compound (CAS 161363-17-3) at 14 μM show a strong rise after 40 minutes. At 28 μM, the lead compound has an even more pronounced effect rising to 200 after 30 minutes. The inhibition of HSP27 correlates with the affinity of the respective small molecule with the binding pocket of HSP27. The light scattering assay does not provide quantitative measurement regarding the amount of protein aggregates. Therefore, we carried out a centrifugation-based assay to quantify the client protein citrate synthase in precipitates. Table 3 summarizes the results of the functional assay by quantifying the amount of precipitated citrate synthase in the presence of HSP27 and compares the inhibitory effect of lead compounds with BVDU. All six leads effectively inhibit HSP27′s chaperone activity. Comparing to BVDU, there is an improved HSP27 inhibition of roughly 50–80 times.

**Table 3:** All lead compounds inhibit HSP27′s chaperone activity significantly better than BVDU

CAS Nr.	Dosage	Relative CS Precipitation	Relative Inhibition
50-53-3	10 μM	1,06	80 ×
61-00-7	10 μM	0,99	74 ×
104715-80-2	10 μM	0,92	69 ×
161363-17-3	10 μM	0,71	53 ×
1222812-38-5	10 μM	0,65	49 ×
1222781-87-4	10 μM	0,81	61 ×
53-86-1	–	–	–
69304-47-8	750 μM	1	1 ×

### All lead compounds significantly reduce drug resistance

Numerous failures in chemotherapy are due to the rapid resistance development of cancer cells to cytostatic therapy. Resistant cancer cells produce more HSP27, which inhibits apoptotic proteins, thus retarding their demise and creating resistance. Bortezomib is a cytostatic whose HSP27-driven development of resistance is well documented [10]. Multiple myeloma cell line, RPMI-8226, was selected because Bortezomib is an FDA approved therapeutic in this cancer. RPMI-8226 cells respond to Bortezomib, express HSP27, and develop Bortezomib-resistance. Additionally, we considered the lymphoma cell line U-937, to show the effects in a different type of cancer.

To test resistance development, cells were seeded at a density of 100,000 cells/ml and serially passaged. The dose of Bortezomib was increased each passage. Test compounds were dosed between 0.5 and 1 μM. In comparison, the positive control BVDU was dosed at 30 μM to show a similar effect.

Figure 3B shows a treatment using 0.75 *μ*M acepromazine, which significantly inhibits resistance formation towards Bortezomib. Only 30,000 cells/ml medium remained after three passages of acepromazine treatment. In comparison, 160,000 cells/ml remained using 30 μM BVDU and 350,000 cells/ml remained for the Bortezomib-only treatment without HSP27 inhibitor. Thus, acepromazine caused a tenfold reduction in living cells compared to Bortezomib-only. In comparison to BVDU, acepromazine leaves 5 times less living cells at a 40 times lower concentration.

Table 4 summarizes the results on drug resistance for all compounds. All lead compounds reduce resistance development significantly at much lower concentrations than BVDU. Detailed graphs can be found in the Supplementary, Figure S4. Table 1 summarizes the results on binding, inhibition of chaperone function, and inhibition of resistance development for all compounds.

**Table 4:** All lead compounds reduce drug resistance significantly

Cell line	CAS Nr.	Dosage	Dosage (relative to BVDU)	Cell Count × 10^3^/ml		
				Bortezomib only	Bortezomib plus BVDU	Bortezomib plus Compound
RPMI-8226	50-53-3	0,5 μM	1/60	350	160	70
RPMI-8226	61-00-7	0,75 μM	1/40	350	160	30
RPMI-8226	104715-80-2	1 μM	1/30	950	290	240
RPMI-8226	161363-17-3	1 μM	1/30	950	290	270
RPMI-8226	1222812-38-5	1 μM	1/30	510	340	290
RPMI-8226	1222781-87-4	1 μM	1/30	510	340	380
U-937	61-00-7	0,75 μM	1/40	450	250	70
U-937	104715-80-2	0,75 μM	1/40	450	250	30

One lead, acepromazine, performs 5-6 fold better than BVDU (30 μM) at one fortieth of the dosage.

### All lead compounds are non-toxic

The positive effect reported above, could be simply due to the toxicity of the lead compounds. If this was true, the lead compounds would show this positive effect independent of the co-treatment with the cytotoxic drug. As a consequence, the lead compounds would not be suitable as co-treatment in cancer therapy. To address this problem, we ran the resistance assay without cytotoxic treatment. I.e. we compared the cell growth of untreated cells to cells treated with one of the lead compounds on its own. As a control, we also tested BVDU on its own. These results are summarized in Supplementary Figure S5. Over the course of two to four weeks, there is no difference in cell growth between untreated and treated cells in the control group. This means, that the lead compounds and also BVDU only are not toxic. They unfold their effect of reducing cell growth only together with the cytotoxic treatment and thus act in synergy. Furthermore, two of the lead compounds are already in use in animals and in humans, respectively (see discussion below), which implies that they are also non-toxic *in vivo*.

## DISCUSSION

### HSP27 inhibitors are rare

Despite HSP27′s importance as cancer target, there are – to the best of our knowledge – currently only two cancer therapy approaches targeting HSP27 under clinical investigation. One is the experimental antisense oligonucleotide Apatorsen (OGX-427) by Oncogenex. Apatorsen has been investigated in several clinical trials in different tumor types according to the U. S. National Institute of Health (clinicaltrials.gov). Chi *et al*. published promising preliminary results in a phase 1 dose finding study using OGX-427 (clinicaltrials.gov ID NCT00487786). The authors reported decreased tumor markers and less circulating tumor cells in castration resistant prostate cancer and other advanced cancers [32]. Squamous cell lung cancer patients are currently recruited to study Apatorsen in combination with Gemcitabine and Carboplatin. Lelj-Garolla *et al*. observed erlontinib-resistance related to increased HSP27 levels in HCC827 and A549 NSLC cell lines and presented preclinical evidence that inhibition of HSP27 using OGX-427 in combination with erlontinib treatment or cytotoxic drugs enhanced antitumor activity and delayed A549 xenograft growth in mice [33].

The second approach relates to BVDU, which forms the core of our drug repositioning pipeline. BVDU directly binds HSP27 and showed promising results in an *in vivo* study on rats and a phase I study with 20 patients [25]. However, a phase II clinical trial did not lead to consistent results. Since the six compounds put forward in this paper are chemically distinct from BVDU they may overcome the problems leading to inconsistent results for BVDU.

### Model of HSP27

At the heart of this study is a structural model of HSP27. Roughly half of HSP27 consists of a stable alpha-crystallin domain and half of an unstable unstructured region. The alpha-crystallin domain, which is highly conserved [34], was crystalized at 2.2A resolution [35], but the unstable region eludes crystallization. HSP27 models – including our own [25] - have been put forward [36], but are questionable due to the instability of the unstructured region. Unfortunately, the predicted binding site [25] is in the unstructured region. Even worse, it comprises the flexible terminus. However, Heinrich *et al*. showed that mutating one of the two key phenylalanines does not prevent BVDU binding and mutating both does. The results presented in this paper give further support that the originally identified binding site is correctly predicted. It remains an interesting open question how the predicted binding site relates to oligomerization interfaces of HSP27.

### Docking of lead binders

Recently, Irwin *et al*. summarized how docking can be used in drug repositioning [37]. The authors argue that such an *in silico* approach is on a par with experimental high-throughput screening. Additionally, it can be scaled up by increasing computing power and it only requires models of the ligands, but not the ligand itself as substance. Here, we pursue such a docking approach to reduce the original ligand list of 228 to 29. Ligand docking has to be considered carefully as *in silico* and experimental poses and binding affinities often disagree. As a consequence, Irwin *et al.* mention hit rates of 10% over large libraries [37]. Therefore, a key contribution of this paper is the generation of a very small, targeted library of chemically diverse compounds, which led to a hit rate of 100%. The crucial step for the small size of the library was the exploitation of the shared binding site.

For docking, we chose AutoDock, as it is very widely used with competitive performance [38]. We evaluated AutoDock's scores for the TK binding site against experimental affinities obtained from BindingDB [27] and found a weak correlation of 0.31 (see Supplementary Figure S3A). This means that it is likely that the 199 compounds with lower docking scores than BVDU may still contain good inhibitors. However, for this analysis they were not considered. We also manually inspected AutoDock poses for the selected compounds and found e.g. that acepromazine uses pi-stacking in the AutoDock pose for VTK and for HSP27 (see Supplementary Figure S3B). This preservation of a molecular interaction type further supports the high scores of acepromazine and the positive experimental results. At the same time, it must be noted that pi-stacking on its own is a very general interaction pattern and many binding sites in the PDB use pi-stacking. A detailed definition of the key interactions defining BVDU binding to VTK beyond pi-stacking is therefore a promising screening approach for new compounds beyond TK binders.

On a more general note, docking the small and targeted compound library derived from the knowledge of the remotely similar binding site of HSP27 and VTK, is a key step for the successful.

### Old drugs, new drugs

Drug repositioning has the advantage of generating new features for known compounds, which are more easily available and which are characterized already. However, they may not be the optimal inhibitors. For the compounds we identified it may be promising to identify common structural patterns and to derive from these patterns novel compounds with even more advantageous binding properties. As mentioned in the results, our analysis shows that BVDU's bromovinyl group is not essential for HSP27 inhibition and that a number of different linkers and ring structures are possible opening up many avenues for future improvements.

### New classes of inhibitors not related to BVDU

A key contribution of this paper is a change in perspective for drug development. Starting from the lead compound BVDU we did not generate and test derivatives of BVDU, but instead we uncovered the binding mechanism and exploited the detour via a remote binding site similarity between HSP27 and VTK to generate lead compounds. These leads are largely independent of BVDU and while they are mostly nucleoside analogues they also cover very different classes such as furo-pyrimidinones and the phenothiazine acepromazine. This approach has the additional advantage that it maps out a larger compound space covering more and different chemical properties besides the target binding. This approach to repositioning is general and can be applied to any scenario where suitable structural data is available.

### Chlorpromazine in cancer

Acepromazine emerged as a compound that ranked highly in the *in silico* pipeline and proved successful in all three experimental assays covering binding, function, and resistance development. Since acepromazine is used in animals, we also investigated its twin chlorpromazine used in humans. Chlorpromazine is also a phenothiazine with antipsychotic effect. It blocks postsynaptic dopamine receptors in cortical and limbic areas of the brain and in the chemical trigger zone. The former reduces hallucinations and delusions, while the latter is used as a sedative in animals and appears to exert antiemetic activity [39]. Chlorpromazine has also been used as antipsychotic drug to treat pain and side effects in advanced cancer [40]. There are reports dating back over 40 years, which suggest that psychiatric patients taking chlorpromazine have lower incidence of breast cancer [41]. Recently, Yde *et al*. corroborated this observation by showing that chlorpromazine enhances the cytotoxic effect of tamoxifen in tamoxifen-resistant human breast cancer cells [42]. The beneficial effect has also been found in colorectal cancer [43].

### HSP27 as a therapeutic target independent of chemotherapy

While we focus on HSP27 inhibition as a co-treatment to cytotoxic drugs, there is evidence emerging that down-regulation of HSP27 - independent of cytotoxic compounds - induces long-term dormancy of angiogenic breast cancer cells [44]. Consistently, up-regulation of HSP27 in previously non-angiogenic cells induced expansive tumor growth *in vivo*. The study was carried out in a mouse model. These results show that the compounds put forward here may not only act as co-treatment, but may have the potential as cancer drugs on their own.

### Understanding chaperone function

It is an open question how small heat shock proteins function as chaperones. The tertiary and quaternary structures of HSP27 are important to answer this question. While the structure of the highly conserved alpha-crystallin domain is solved [34, 35], there is no tertiary or quaternary structure of full length HSP27. For the latter, Stengel *et al*. [45] investigate the dynamics of oligomerization and found a large number of heterogeneous stoichiometries, which – they argue – is an integral part of HSP function. The compounds we identified inhibit HSP27′s function as chaperone. Thus, they may provide valuable information to advance the understanding of the dynamic oligomerization process observed by van Montfort *et al.* [34].

### Alternative methods to tackle difficult target proteins

Some proteins are very interesting targets because of their biomedical relevance, however, it is difficult to apply common methods in drug discovery to identify and characterize inhibitory compounds. In case of the small molecular chaperone HSP27, probably because of its intrinsic structural dynamics, no crystal structure of full-length protein is available. Because of the lack of method to quantify protein aggregates, no high throughput assay has been established for drug screening campaigns. Moreover, its promiscuous binding to various client proteins also causes its non-specific interaction with biosensor chip surface, thus it is difficult for conventional on chip validation method (e.g. surface plasma resonance and interferometry). In this work, in addition to modeling the protein structure and identifying lead inhibitors using an *in silico* approach, we have applied speed-screening and centrifugation-based precipitation quantification assay to validate the protein-ligand interaction in solution. The experimental characterization has not only confirmed the binding property from *in silico* prediction, but also paved the way for designing a high throughput screening campaign against HSP27 chaperone activity.

## MATERIALS AND METHODS

### VTK and non-VTK compounds

The Uniprot query thymidine and kinase and reviewed:yes and organism:”Human herpesvirus” returned 13 herpes thymidine kinase sequences. The drug-target databases BindingDB, DrugBank, PDB, and TTD returned 115 compounds binding to those. BindingDB provided affinity scores for 83 of them. Similarly, the query thymidine and kinase and reviewed:yes and not organism:”Human herpesvirus” returned 241 results (both queries were run on 30/05/2011), for which there were twelve structures in the PDB. Eight of these non-viral thymidine kinases have a binding site similar to the BVDU binding site of the viral thymidine kinase in PDB ID 1OSN. Similarity was defined through the structure comparison tool SMAP [46] obtaining a *p-value* of 10^−3^ or better. For these eight non-viral TKs BindingDB, DrugBank, PDB, and TTD list 113 compounds. BindingDB provided affinity scores for 41 of them.

### Docking

The compounds were docked to the viral thymidine kinase PDB ID 1OSN [47] and the model of HSP27 used in [25] with AutoDock 4.2 (rigid body docking) [48]. AutoDockTools (ver.1.5.4) was used to add polar hydrogen partial charges and Autodock atom types. AutoGrid 4 was used to build two grid boxes (66 × 60 × 60 A) and (50 × 60 × 50 A) with 0.375 A spacing for the respective proteins. The docking calculations were performed using the Lamarckian genetic algorithm procedure with the following parameters: a maximum number of energy evaluations (evals 5,000,000), an initial population of 150 randomly placed individuals, a maximum number of 27,000 generations, a mutation rate of 0.02, a cross-over rate of 0.80 and an elitism value (number of top individuals that automatically survive) of 1. For the local search, the Soils and Wets algorithm was applied to a maximum of 300 iterations per search. Thirty independent docking runs were carried out for each compound. Using Autodock 4, compound poses were clustered based on their Root Mean Square Deviation (RMSD) values. Finally, the change in Gibbs energy (ΔG) is estimated by Autodock and used to calculate binding affinities with the following equation: K_i_ = e ^−ΔG / R T^.

### Chemical similarity

Chemical compound similarity was computed with PubChem: pubchem.ncbi.nlm.nih.gov/assay/assay.cgi?p=clustering. Compounds are represented as fingerprints of chemical features. The similarity is expressed as Tanimoto coefficient, i.e. the number of shared features divided by the number of the union of features. A Tanimoto score of 0.9 or better is considered similar.

### Compounds

CAS No. 1222812-38-5 and CAS No. 1222781-87-4 were gifts from María Jesús Pérez Pérez, Instituto de Química Médica (CSIC), Madrid, Spain. CAS No. 61-00-7, CAS No. 50-033-3, and CAS No. 53-86-1 (negative control) were obtained from Sigma. CAS No. 161363-17-3 and CAS No. 104715-80-2 were gifts from George Wright, GLS Synthesis, Worcester, MA, USA. CAS No. 69304-47-8 was a gift from Rudolf Fahrig, RESprotect, Dresden, Germany.

### Expression and purification of HSP27 recombinant protein

To test the predicted HSP27 inhibitors HIS-tagged recombinant HSP27 protein was synthesized using the E. coli BL21(DE3) strain. The HSP27 expression plasmid was built upon the pRSET A expression vector (Invitrogen). The HSP27 DNA sequence was codon optimized for expression in E. coli. Recombinant HSP27 protein was purified using HIS-traps and subsequently Superdex75 columns (GE Healthcare) according to the manufacturer's instructions.

### Binding assay: SpeedScreen

The SpeedScreen [49] uses Sephadex columns, which let bigger molecules (compound bound to HSP27) pass and smaller (less than 5000g/Mol) ones (compound not bound to HSP27) not. We used PD G-25 SpinTraps (GE Healthcare). The compounds were incubated with HSP27 in 40 mM Hepes, pH 7,4 for 10 min at room temperature and loaded in an appropriate volume of the mixture on a PD G-25 SpinTrap. In parallel another Spin Trap was loaded with the same test compound without the protein. After centrifugation the eluates were analyzed by MS/MS to see if the compounds were carried through the spin traps. Unbound compounds should be retained by the Sephadex G-25 Spin Trap. A Waters ACQUITY TQD System was used for characterization and detection. MS/MS methods for the respective test compounds were developed using the IntelliStart™ Software in order to enhance sensitivity and selectivity. Compounds that were carried through the Sephadex G-25 Spin Traps by HSP27 were detected using LC-ESI-MSMS.

### Functional assay

To test how the test compounds affect the chaperone activity of HSP27, we measured misfolding and aggregation of citrate synthase, a well-known *in vitro* client protein of HSP27. Citrate synthase misfolds and aggregates when exposed to heat shock conditions at 43°C. This heat denaturation is prevented or delayed by the presence of HSP27 [50]. The effectiveness of the test compounds can thus be measured by inhibition of the HSP27 chaperone function. To determine the influence on the aggregation behavior of citrate synthase (CS) at 43°C we used 1.44 μM CS, 481 nM HSP27 (HSP) in a 40 mmol / L HEPES buffer (pH 7.4) and provided BVDU (750 μM) as a positive control and various concentrations of the respective new HSP27 inhibitor (typically 10 μM). The samples were incubated at 43°C and the aggregation behavior of CS was monitored in a spectrometer (PerkinElmer LS55) at a wavelength of 500 nm.

### Capillary electrophoresis of CS precipitates

The heat-denatured protein aggregates produced by the aggregation assay are water-insoluble and can be separated from the supernatant by centrifugation at 16.000 × g for 10 min at room temperature. Precipitated proteins (CS and HSP27) were visualized and quantified using capillary electrophoresis (Beckman PA800). The amount of precipitated CS is an indirect measure for the efficiency of the respective HSP27 inhibitor. The relative amount of precipitated citrate synthase was normalized over a defined amount of BSA as an internal standard. The relative amount of citrate synthase precipitated in the presence of 750 μM BVDU was set equal to 1. The other test substances were used at a concentration of 10 μM.

### Resistance assay

RPMI-8226 cells, a human multiple myeloma cell line, were obtained from DSMZ (Leibniz Institute DSMZ-German Collection of Microorganisms and Cell Cultures). Cells were grown in RPMI 1640 medium (FG 1235; Biochrom AG, Berlin, Germany) supplemented with 10% (v/v) heat-inactivated fetal bovine serum in a humidified atmosphere containing 5% CO_2_ at 37°C. Logarithmically growing cells were seeded at a density of 100,000 cells/ml and incubated with Bortezomib (Velcade) in combination with or without HSP27 inhibitors. Cells were periodically counted using C-Chip Neubauer-improved counting chambers (Peqlab) and serially passaged. Cells were always kept below 1,000,000 cells per ml and typically passaged when cell count reached 800,000 cells per ml. The starting dose of Bortezomib was typically 0.1 ng/ml. The dose of Bortezomib was increased each passage, e.g.: 1^st^ passage 0.1 ng/ml Bortezomib, 2^nd^ passage 0.2 ng/ml Bortezomib, 3rd passage 0.3 ng/ml Bortezomib.

In parallel cells were treated with Bortezomib plus 30 μM BVDU as a positive control and with Bortezomib plus the respective HSP27 compound (typically 1 μM or lower). The dose of BVDU and the new HSP27 inhibitors remained unchanged throughout the experiment. Untreated cells, BVDU-only, and test compound-only treatments served as controls. Treatment with 30 μM BVDU or 0.5–1 μM of the respective HSP27 inhibitor test compound did not influence cell growth. The dose of the test compounds was defined in dose finding experiments beforehand to determine the functional but non-toxic dose. U-937 cells (histiocytic lymphoma) were treated as described above for the RPMI-8226 cell line.

## CONCLUSIONS

In conclusion, the heat shock protein HSP27 is overexpressed in many cancers and associated with resistance development against cytotoxic drugs. Thus, inhibitors of HSP27 may improve cancer chemotherapy as co-treatment together with cytotoxic drugs. This paper presents three key contributions: First, exploiting a shared binding site of HSP27 and VTK we were able to generate a very small and targeted library for *in silico* docking, which led to a 100% success rate in the subsequent experimental validation. The principle is general and can be applied to other drug discovery tasks. Second, we identified and validated six lead compounds that are significantly better binders and functional modulators of HSP27 than currently known HSP27 inhibitors. Furthermore, these inhibitors require much lower doses, which will be an important advantage in future experiments in animals and in humans. The key contribution relates to the chemical diversity of the identified compounds. Because, although the original compound library for the *in silico* screening was small, it did not just contain derivatives of the known HSP27 binder BVDU, but covered instead a wide variety of chemically very different compounds. Together with the experimental validation, this means that the novel HSP27 inhibitors map out the space of possible inhibitors and shed more light on the true mode of action for HSP27 inhibition. In particular, these findings mean that bromovinyl group of BVDU is not necessary for HSP27 inhibition. An important aspect for future improvement of cancer therapy is, that the compounds are not toxic. Our results show that the lead compounds suppress resistance development only in combination with the cytotoxic treatment and show no effect on their own. Additionally, two of the compounds are in use in animals and humans, respectively, which means they are non-toxic *in vivo*, too. The third contribution relates to the assays developed. Heat shock proteins are important and so is their inhibition. The established assays are novel for heat shock proteins and shed light onto binding, function, and resistance suppression. Especially, the functional assay is suitable for higher throughput and may be taken up by others to improve our understanding of chaperone function and cancer.

In a nutshell, docking with a targeted compound library led to six novel compounds, which suppress chemoresistance in cancer cells.

## SUPPLEMENTARY MATERIALS FIGURES


