# Supplementary material for: New HSP27 inhibitors efficiently suppress drug resistance development in cancer cells
Source: Oncotarget. 2016 Sep 8;7(42):68156–69. doi: 10.18632/oncotarget.11905 (PMC5356546; doi:10.18632/oncotarget.11905)
Supplement: Supplementary file 1 [file oncotarget-07-68156-s001.pdf]

New HSP27 inhibitors efficiently suppress drug resistance development in cancer cells

Supplementary Materials

PubchemID	Rank	HSP27	TK
CID44333373	1	-5,74	-5,83
CID44333745	2	-5,27	-4,65
CID46884565	3	-5,08	-4,70
CID6077	4	-5,00	-4,90
CID3031263	5	-4,93	-4,80
CID46197457	6	-4,92	-4,83
CID3007762	7	-4,84	-5,47
CID46885302	8	-4,75	-4,94
CID46846243	9	-4,71	-4,63
CID44333760	10	-4,59	-4,68
CID6319949	11	-4,55	-4,59
CID44333390	12	-4,51	-5,17
CID16756295	13	-4,50	-5,25
CID24750304	14	-4,41	-4,85
CID46885301	15	-4,38	-4,58
CID46197456	16	-4,37	-5,22
CID16121720	17	-4,27	-4,50
CID46888108	18	-4,14	-4,52
CID46884567	19	-4,11	-5,20
CID46884566	20	-4,00	-4,80
CID44420579	21	-3,93	-5,14
CID44420577	22	-3,78	-4,65
CID6439190	23	-3,57	-5,03
CID11675456	24	-3,36	-4,48
CID9906078	25	-3,27	-4,48
CID387676	26	-3,21	-4,49
CID446725	27	-3,17	-4,99
CID333502	28	-3,10	-4,79
CID6505368	29	-2,56	-4,48

Note
Yellow Experimentally tested compounds
Rank is sorted by HSP27 computed Ki
HSP27 and TK computed docking Ki on log 10 scale

Supplementary Figure S1: Compound, computational binding affinity (log 10 Ki value) against HSP27 and TK for 29 compounds, which have better values than BVDU on both targets.

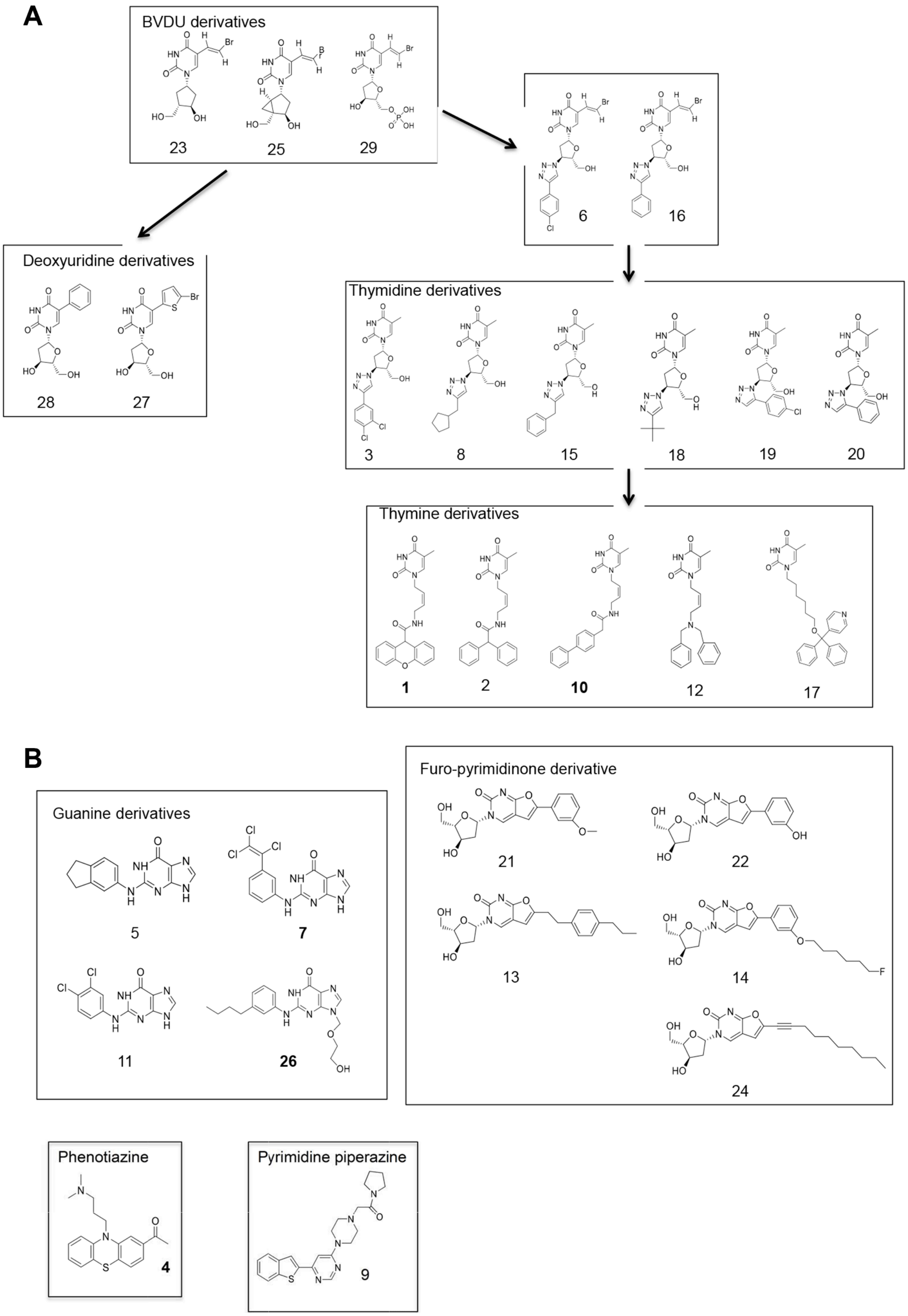

Supplementary Figure S2: Structural relation of compounds. (**A**) Compounds, which can be related to BVDU. (**B**) Compounds, which are independent of BVDU. Compounds 1, 4, 7, 10, 26 marked in bold were selected for experimental validation.

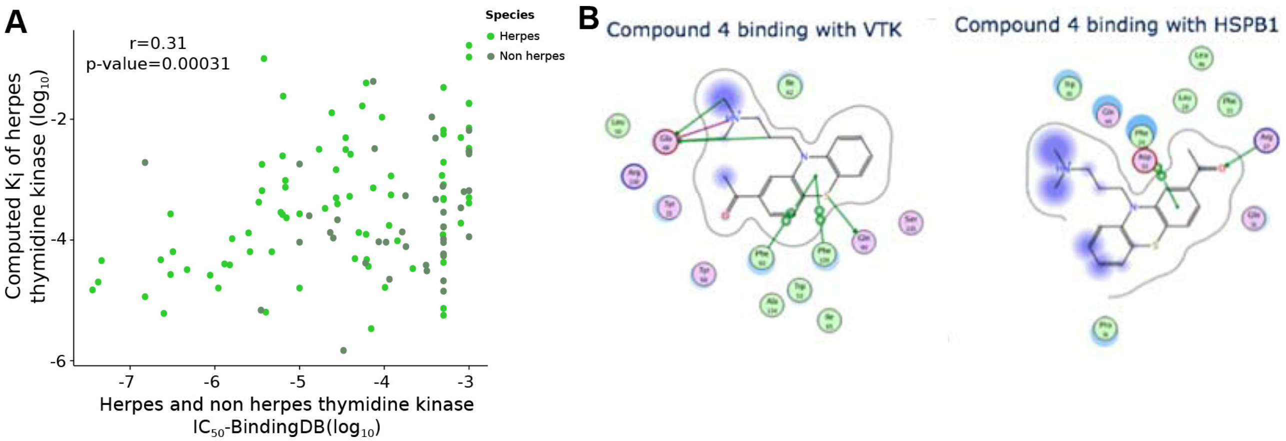

Supplementary Figure S3: (**A**) Computational Autodock Ki values for TK versus experimental binding affinities from BindingDB. A weak correlation shows that docking is viable, but results have to be considered carefully. (**B**) Computational docking of acepromazine against HSP27 and TK preserves key binding feature of pi-stacking.

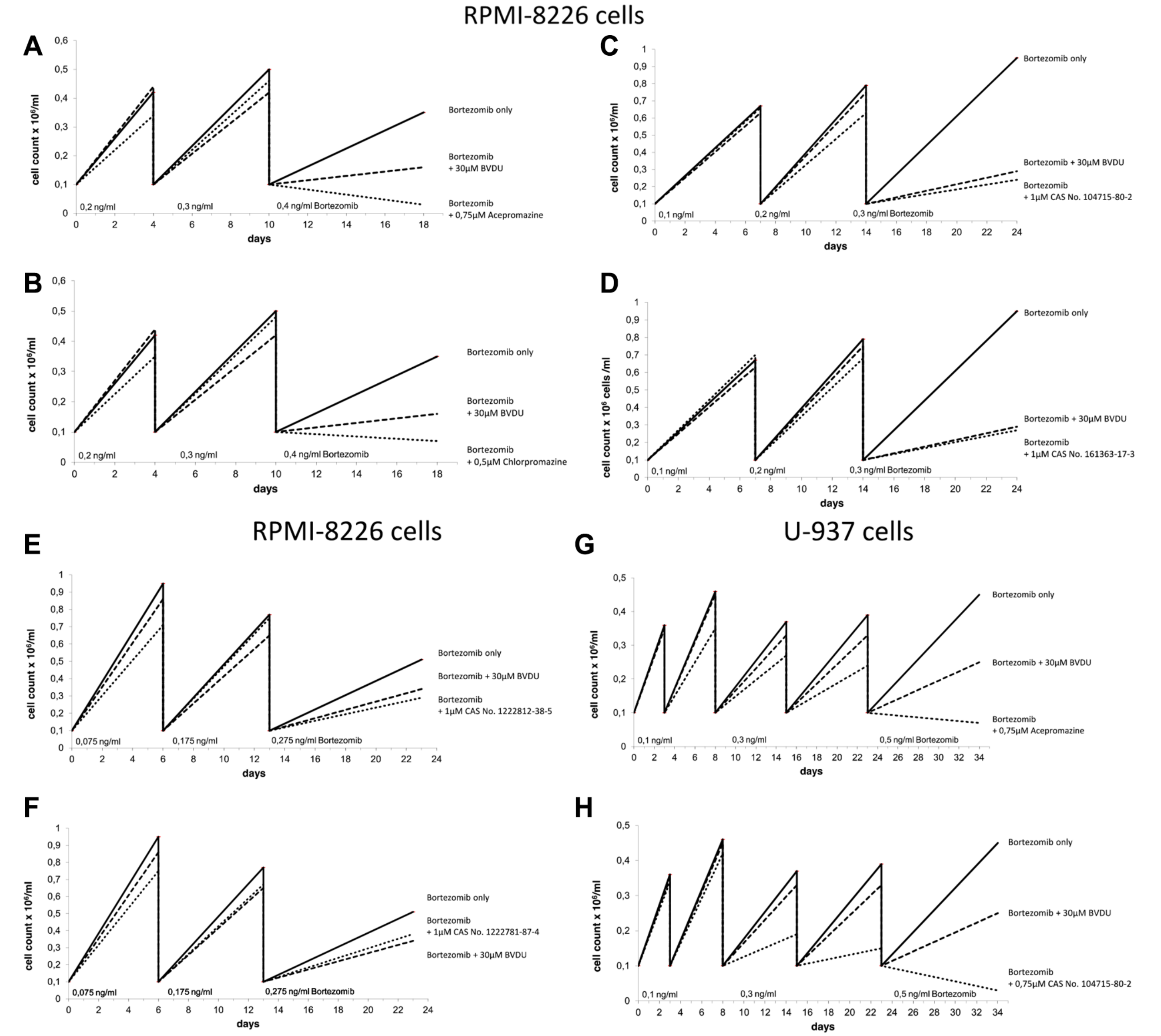

Supplementary Figure S4: Growth curves of Bortezomib only, Bortezomib plus BVDU, and Bortezomib plus compound show that compounds reduce resistance development in cancer cells significantly.

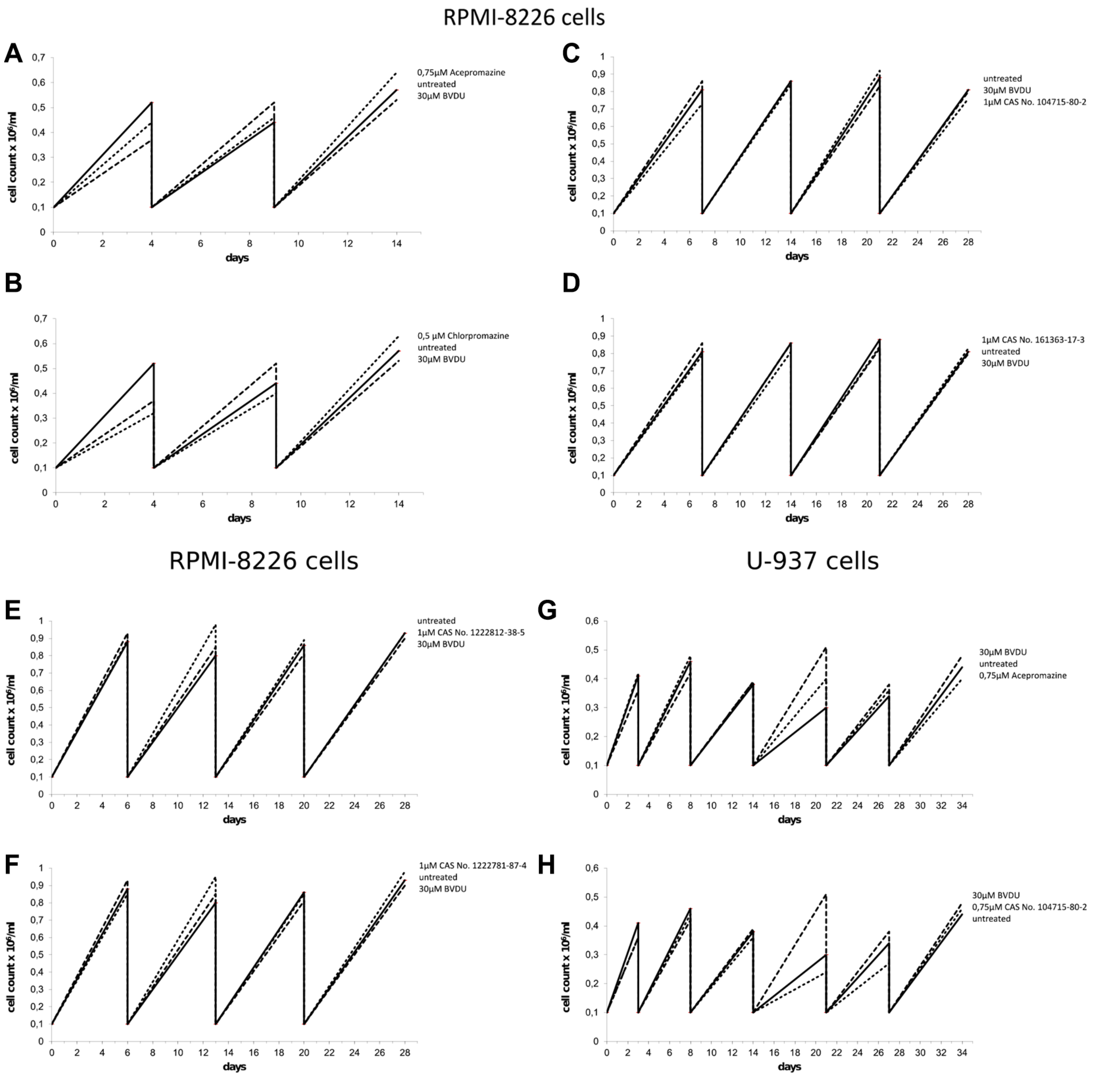

Supplementary Figure S5: Growth curves without cytostatic treatment show that compounds alone do not influence cell growth.
